# Completed Local Ternary Pattern for Rotation Invariant Texture Classification

**DOI:** 10.1155/2014/373254

**Published:** 2014-04-07

**Authors:** Taha H. Rassem, Bee Ee Khoo

**Affiliations:** School of Electrical & Electronic Engineering, Universiti Sains Malaysia, Engineering Campus, Nibong Tebal, 14300 Penang, Malaysia

## Abstract

Despite the fact that the two texture descriptors, the completed modeling of Local Binary Pattern (CLBP) and the Completed Local Binary Count (CLBC), have achieved a remarkable accuracy for invariant rotation texture classification, they inherit some Local Binary Pattern (LBP) drawbacks. The LBP is sensitive to noise, and different patterns of LBP may be classified into the same class that reduces its discriminating property. Although, the Local Ternary Pattern (LTP) is proposed to be more robust to noise than LBP, however, the latter's weakness may appear with the LTP as well as with LBP. In this paper, a novel completed modeling of the Local Ternary Pattern (LTP) operator is proposed to overcome both LBP drawbacks, and an associated completed Local Ternary Pattern (CLTP) scheme is developed for rotation invariant texture classification. The experimental results using four different texture databases show that the proposed CLTP achieved an impressive classification accuracy as compared to the CLBP and CLBC descriptors.

## 1. Introduction

Nowadays, texture analysis and classification have become one of the important areas of computer vision and image processing. They play a vital role in many applications such as visual object recognition and detection [1, 2], human detector [3], object tracking [4], pedestrian classification [5], image retrieval [6, 7], and face recognition [8, 9].

Currently, many textures feature extraction algorithms that have been proposed to achieve a good texture classification. Most of these algorithms are focusing on how to extract distinctive texture features that are robust to noise, rotation, and illumination variance. These algorithms can be classified into three categories [10]. The first category is the statistical methods such as polar plots and polarograms [11], texture edge statistics on polar plots [12], moment invariants [13], and feature distribution method [14]. The second category is the model based methods such as simultaneous autoregressive model (SAR) [15], Markov model [16], and steerable pyramid [17]. The third category is the structural methods such as topological texture descriptors [18], invariant histogram [19], and morphological decomposition [20]. All of these algorithms as well as many other algorithms are reviewed briefly in many review papers [10, 21, 22].

**Figure 1:**
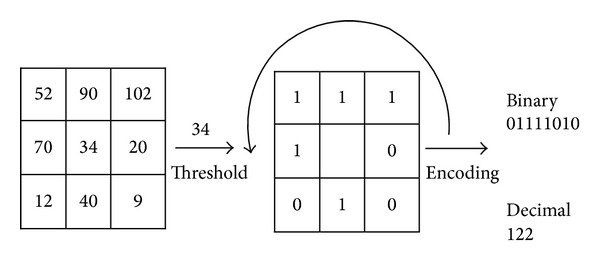
LBP operator.

Local Binary Pattern (LBP) operator was proposed by Ojala et al. [23] for rotation invariant texture classification. It has been modified and adapted for several applications such as face recognition [8, 9] and image retrieval [7]. The LBP extraction algorithm contains two main steps, that is, the thresholding step and the encoding step. This is shown in Figure 1. In the thresholding step, all the neighboring pixel values in each pattern are compared with the value of their central pixel of the pattern to convert their values to binary values (0 or 1). This step helps to get the information about the local binary differences. Then in the encoding step, the binary numbers obtained from the thresholding step are encoded and converted into a decimal number to characterize a structural pattern. In the beginning, Ojala et al. [24] represented the texture image using textons histogram by calculating the absolute difference between the gray level of the center pixel of a specific local pattern and its neighbors. Then the authors proposed the LBP operator by using the sign of the differences between the gray level of the center pixel and its neighbors of the local pattern instead of magnitude [23]. LBP proposed by Ojala et al. [23] has become the research direction for many computer vision researchers. This is because it is able to distinguish the microstructures such as edges, lines, and spots. The researchers aim to increase the discriminating property of the texture feature extraction to achieve impressive rotation invariant texture classification. So, many of the variants of the LBP have been suggested and proposed for rotation invariant texture classification. The center-symmetric Local Binary Pattern (CS-LBP) proposed by Heikkil et al. [25] is an example for that. Unlike the LBP, they compared center-symmetric pairs of pixels to get the encoded binary values. Liao et al. [26] proposed Dominant LBP (DLBP) by selecting the dominant patterns from all rotation invariant patterns. Tan and Triggs [27] presented a new texture operator which is more robust to noise. They encoded the neighbor pixel values into 3-valued codes (−1,0, 1) instead of 2-valued codes (0,1) by adding a user threshold. This operator is known as a Local Ternary Pattern (LTP). Guo et al. [28] combined the sign and magnitude differences of each pattern with all central gray level values of all patterns to propose a completed modeling of LBP, called completed LBP (CLBP). Khellah [29] proposed a new method for texture classification, which combines Dominant Neighborhood Structure (DNS) and traditional LBP. Zhao et al. [30] proposed a novel texture descriptor, called Local Binary Count (CLBC). They used the thresholding step such as in LBP. Then they discarded the structural information from the LBP operator by counting the number of value 1's in the binary neighbor sets instead of encoding them.

**Figure 2:**
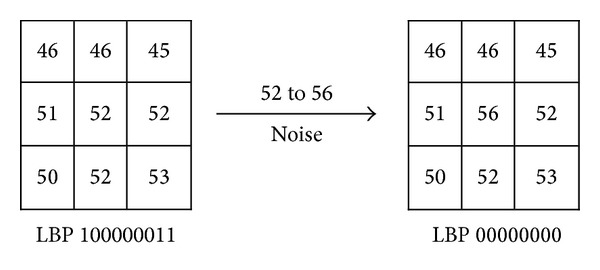
The example for LBP operator's noise sensitivity.

**Figure 3:**
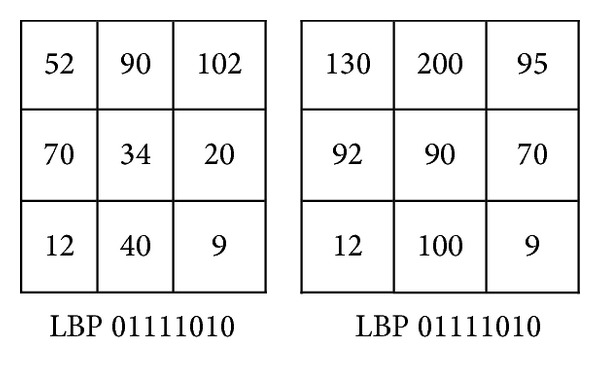
Similar LBP codes for two different texture patterns.

Although, some LBP variant descriptors such as CLBP and CLBC have achieved remarkable classification accuracy, they inherit the LBP weaknesses. The LBP suffers from two main weaknesses. It is sensitive to noise and sometimes may classify two or more different patterns falsely to the same class as shown in Figures 2 and 3. The LTP descriptor is more robust to noise than LBP. However, the latter weakness may appear with the LTP as well as with LBP.

In this paper, we are enhancing the LTP texture descriptor to increase its discriminating property by presenting a completed modeling for LTP operator and proposing an associated completed Local Ternary Pattern (CLTP). The experimental results illustrate that CLTP is more robust to noise, rotation, and illumination variance, and it achieves higher texture classification rates than CLBP and CLBC. The rest of this paper is organized as follows.  Section 2 briefly reviews the LBP, LTP, CLBP, and CLBC.  Section 3 presents the new CLTP scheme. Then, in Section 4, the experimental results of different texture databases are reported and discussed. Finally, Section 5 concludes the paper.

## 2. Related Work

In this section, a brief review of the LBP, LTP, CLBP, and CLBC is provided.

### 2.1. Brief Review of LBP

As shown in Figure 1, the LBP operator is computed for the center pixel by comparing the intensity value of it with the intensity values of its neighbors. This process can be expressed mathematically as follows:

(1) $$\begin{array}{c} {\text{LBP}}_{P,R}=\overset{P-1}{\underset{p=0}{\sum }}2^{p}s\left(i_{p}-i_{c}\right),\;\;s\left(x\right)=\{\begin{array}{cc} 1, & x\geq 0,\\ 0, & x<0, \end{array} \end{array}$$

where *i*_*c*_ and *i*_*p*_ (*p* = 0,…, *P* − 1) denote the gray value of the center pixel and gray value of the neighbor pixel on a circle of radius *R*, respectively, and *P* is the number of the neighbors. Bilinear interpolation estimation method is used to estimate the neighbors that do not lie exactly in the center of the pixels. LBP_*P*,*R*_^ri^ and LBP_*P*,*R*_^riu2^ are rotation invariant of LBP and uniform rotation invariant of LBP, respectively. These two enhanced LBP operators are proposed by Ojala et al. [23].

After completing the encoding step for any LBP operators, that is, LBP_*P*,*R*_^ri^ and LBP_*P*,*R*_^riu2^, the histogram can be created based on the following equation:

(2) $$\begin{array}{c} H\left(k\right)=\overset{I}{\underset{i=0}{\sum }}\;\overset{J}{\underset{j=0}{\sum }}f\left({\text{LBP}}_{P,R}\left(i,j\right),k\right),\;k\in \left[0,K\right],\\ f\left(x,y\right)=\{\begin{array}{cc} 1, & x=y,\\ 0, & \text{otherwise}, \end{array} \end{array}$$

where *K* is the maximal LBP pattern value.

### 2.2. Brief Review of LTP

**Figure 4:**
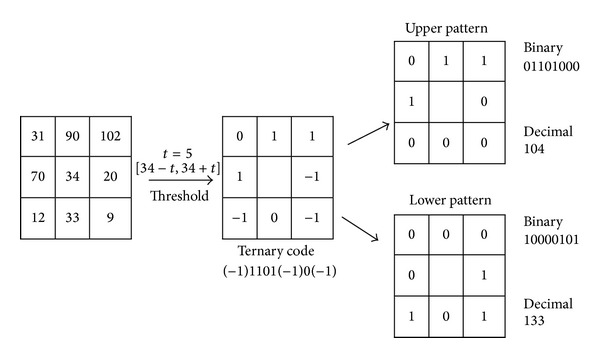
LTP operator.

Tan and Triggs [27] presented a new texture operator which is more robust to noise. The LBP is extended to 3-valued codes (−1,0, 1).  Figure 4 shows an example of LTP operator. The mathematical expression of the LTP can be described as follows:

(3) $$\begin{array}{c} {\text{LTP}}_{P,R}=\overset{P-1}{\underset{p=0}{\sum }}2^{p}s\left(i_{p}-i_{c}\right),\;\;s\left(x\right)=\{\begin{array}{cc} 1, & x\geq t,\\ 0, & -t<x<t,\\ -1, & x<-t, \end{array} \end{array}$$

where *i*_*c*_, *i*_*p*_, *R*, and *P* are defined before in (1) and *t* denotes the user threshold. After thresholding step, the upper pattern and lower pattern are constructed and coded as shown in Figure 4. The LTP operator is the concatenation of the code of the upper pattern and the lower pattern.

### 2.3. Brief Review of Completed LBP (CLBP)

**Figure 5:**
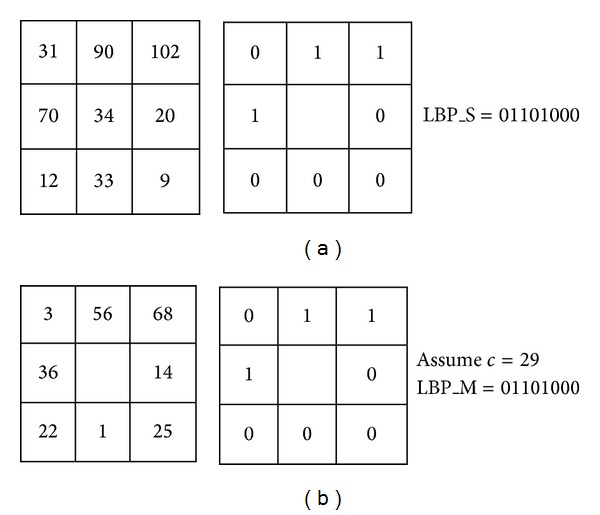
A 3 × 3 sample pattern: (a) sign component (LBP_S code); (b) magnitude components (LBP_M code (assume threshold = 29)).

The completed LBP (CLBP) descriptor was proposed by Guo et al. [28] in order to improve the performance of LBP descriptor. As shown in Figure 5, the image local difference is decomposed into two complementary components: the sign component *s*_*p*_ and the magnitude component *m*_*p*_. Consider the following:

(4) $$\begin{array}{c} s_{p}=s\left(i_{p}-i_{c}\right),\;\;m_{p}=\left|i_{p}-i_{c}\right|. \end{array}$$

Then, the *s*_*p*_ is used to build the CLBP-Sign (CLBP_S), whereas the *m*_*p*_ is used to build CLBP-magnitude (CLBP_M). The CLBP_S and CLBP_M are described mathematically as follows:

(5) $$\begin{array}{c} \text{CLBP\_}{\text{S}}_{P,R}=\overset{P-1}{\underset{p=0}{\sum }}2^{p}s\left(i_{p}-i_{c}\right),\;\;s_{p}=\{\begin{array}{cc} 1, & i_{p}\geq i_{c},\\ 0, & i_{p}<i_{c}, \end{array} \end{array}$$

(6)        CLBP_MP,R=∑p=0P−12pt(mp,c),t(mp,c)={1,|ip−ic|≥c,0,|ip−ic|<c,

where *i*_*c*_, *i*_*p*_, *R*, and *P* are defined before in (1) and *c* in (6) denotes the mean value of *m*_*p*_ in the whole image.

The CLBP_S is equal to LBP, whereas the CLBP_M measures the local variance of magnitude. Furthermore, Guo et al. used the value of the gray level of each pattern to construct a new operator, called CLBP-Center (CLBP_C). The CLBP_C can be described mathematically as follows:

(7) $$\begin{array}{c} \text{CLBP\_}{\text{C}}_{P,R}=t\left(i_{c},c_{I}\right), \end{array}$$

where *i*_*c*_ denotes the gray value of the center pixel and the *c*_*I*_ is the average gray level of the whole image. Guo et al. combined their operators into joint or hybrid distributions and achieved a remarkable texture classification accuracy [28].

### 2.4. Brief Review of Completed LBC (CLBC)

The Local Binary Count (LBC) was proposed by Zhao et al. [30]. Unlike the LBP and all its variants, the authors just counted the number of value 1's of the thresholding step instead of encoding them. The LBC can be described mathematically as follows:

(8) $$\begin{array}{c} {\text{LBC}}_{P,R}=\overset{P-1}{\underset{p=0}{\sum }}s\left(i_{p}-i_{c}\right),\;\;s\left(x\right)=\{\begin{array}{cc} 1, & x\geq 0,\\ 0, & x<0. \end{array} \end{array}$$

**Figure 6:**
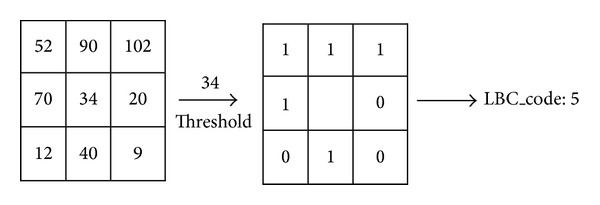
LBC operator.

Similar to CLBP, the authors [30] extended the LBC to completed LBC (CLBC). The CLBC_S, CLBC_M, and CLBC_C were also combined into joint or hybrid distributions and they were used for rotation invariant texture classification. The CLBC_M and CLBC_C can be described mathematically as follows:

(9) $$\begin{array}{c} \text{CLBC\_}{\text{M}}_{P,R}=\overset{P-1}{\underset{p=0}{\sum }}t\left(m_{p},c\right),\;t\left(m_{p},c\right)=\{\begin{array}{cc} 1, & \left|i_{p}-i_{c}\right|\geq c,\\ 0, & \left|i_{p}-i_{c}\right|<c, \end{array}\\ \text{CLBC\_}{\text{C}}_{P,R}=t\left(i_{c},c_{I}\right), \end{array}$$

where *i*_*c*_, *i*_*p*_, *c*, and *c*_*I*_ are defined in (1), (6), and (7). An example of LBC is shown in Figure 6.

In [28], the rotation invariant LBP (LBP_*P*,*R*_^riu2^) is used to construct the CLBP_*P*,*R*_^riu2^. The CLBP_*P*,*R*_^riu2^ is simplified in this paper as CLBP_*P*,*R*_ as well as the proposed CLTP operator.

## 3. Completed Local Ternary Pattern (CLTP)

In this section, we propose the framework of CLTP. Similar to CLBP [28], the LTP is extended to completed modeling LTP (CLTP). As mentioned before, the LTP is more robust to noise than LBP. Furthermore, construct the associated completed Local Ternary Pattern that will help to enhance and increase its the discriminating property. The mathematical model of LTP is shown in (3). In CLTP, local difference of the image is decomposed into two sign complementary components and two magnitude complementary components as follows:

(10) $$\begin{array}{c} s_{p}^{\text{upper}}=s\left(i_{p}-\left(i_{c}+t\right)\right),\;\;s_{p}^{\text{lower}}=s\left(i_{p}-\left(i_{c}-t\right)\right)\;\;\;\;\\ m_{p}^{\text{upper}}=\left|i_{p}-\left(i_{c}+t\right)\right|,\;\;m_{p}^{\text{lower}}=\left|i_{p}-\left(i_{c}-t\right)\right|, \end{array}$$

where *i*_*c*_, *i*_*p*_, and *t* are defined before in (1) and (3).

Then the *s*_*p*_^upper^ and *s*_*p*_^lower^ are used to build the CLTP_S_*P*,*R*_^upper^ and CLTP_S_*P*,*R*_^lower^, respectively, as follows:

(11) $$\begin{array}{c} \text{CLTP\_}{\text{S}}_{P,R}^{\text{upper}}=\overset{P-1}{\underset{p=0}{\sum }}2^{p}s\left(i_{p}-\left(i_{c}+t\right)\right),\\ s_{p}^{\text{upper}}=\{\begin{array}{cc} 1, & i_{p}\geq i_{c}+t,\\ 0, & \text{otherwise}, \end{array}\\ \text{CLTP\_}{\text{S}}_{P,R}^{\text{lower}}=\overset{P-1}{\underset{p=0}{\sum }}2^{p}s\left(i_{p}-\left(i_{c}-t\right)\right),\\ s_{p}^{\text{lower}}=\{\begin{array}{cc} 1, & i_{p}<i_{c}-t,\\ 0, & \text{otherwise}. \end{array} \end{array}$$

The CLTP_S_*P*,*R*_ is the concatenation of the CLTP_S_*P*,*R*_^upper^ and CLTP_S_*P*,*R*_^lower^ as follows:

(12) $$\begin{array}{c} \text{CLTP\_}{\text{S}}_{P,R}=\left[\begin{array}{cc} \text{CLTP\_}{\text{S}}_{P,R}^{\text{upper}} & \text{CLTP\_}{\text{S}}_{P,R}^{\text{lower}} \end{array}\right], \end{array}$$

where *i*_*c*_, *i*_*p*_, *P*, *R*, and *t* in (11) are defined before in (3).

Similar to CLTP_S_*P*,*R*_, the CLTP_M_*P*,*R*_ is built using the two magnitude complementary components *m*_*p*_^upper^ and *m*_*p*_^lower^ as follows:

(13)      CLTP_MP,Rupper=∑p=0P−12pt(mpupper,c),t(mpupper,c)={1,|ip−(ic+t)|≥c,0,|ip−(ic+t)|<c,

(14)      CLTP_MP,Rlower=∑p=0P−12pt(mplower,c),t(mplower,c)={1,|ip−(ic−t)|≥c,0,|ip−(ic−t)|<c,

(15) $$\begin{array}{c} {\text{CLTP\_M}}_{P,R}=\left[\begin{array}{cc} {\text{CLTP\_M}}_{P,R}^{\text{upper}} & {\text{CLTP\_M}}_{P,R}^{\text{lower}} \end{array}\right], \end{array}$$

where *i*_*c*_, *i*_*p*_, *P*, *R*, and *t* in (13) and (14) are defined before in (3) and *c* is defined in (6).

Moreover, the CLTP_C_*P*,*R*_^upper^ and CLTP_C_*P*,*R*_^lower^ can be mathematically described as follows:

(16) $$\begin{array}{c} \text{CLTP\_}{\text{C}}_{P,R}^{\text{upper}}=t\left(i_{c}^{\text{upper}},c_{I}\right),\\ \text{CLTP\_}{\text{C}}_{P,R}^{\text{lower}}=t\left(i_{c}^{\text{lower}},c_{I}\right), \end{array}$$

where *i*_*c*_^upper^ = *i*_*c*_ + *t*, *i*_*c*_^lower^ = *i*_*c*_ − *t* and *c*_*I*_ is the average gray level of the whole image.

The proposed CLTP operators are combined into joint or hybrid distributions to build the final operator histogram like the CLBP and CLBC [28, 30], respectively. In the CLTP, the operators of the same type of pattern; that is, the upper and the lower pattern are combined first into joint or hybrid distributions. Then their results are concatenated to build the final operator histogram. That mean number of bins of CLTP is double in size than the number of bins of CLBP.

## 4. Experiments and Discussion

In this section, a series of experiments are performed to evaluate the proposed CLTP. Four large and comprehensive texture databases are used in these experiments. They are the Outex database [31], Columbia-Utrecht Reflection and Texture (CUReT) database [32], UIUC database [33], and XU_HR database [34]. Empirically, the threshold value *t* is set to 5.

### 4.1. Dissimilarity Measuring Framework

Several metrics are proposed for measuring the dissimilarity between two histograms such as log-likelihood ratio, histogram intersection, and chi-square statistic. Similar to [28, 30], the chi-square statistic is used in this brief. The *χ* ^2^ distance between two histograms *H* = *h*_*i*_ and *K* = *k*_*i*_ where (*i* = 1,2, 3,…*B*) can be mathematically described as follows:

(17) $$\begin{array}{c} {\text{Dissimilarity}}_{\chi^{2}}\left(H,K\right)=\overset{B}{\underset{i=1}{\sum }}\frac{{\left(h_{i}-k_{i}\right)}^{2}}{h_{i}+k_{i}}. \end{array}$$

Furthermore, the nearest neighborhood classifier is used for classification in all experiments in this paper.

### 4.2. Experimental Results on the Outex Database

**Figure 7:**
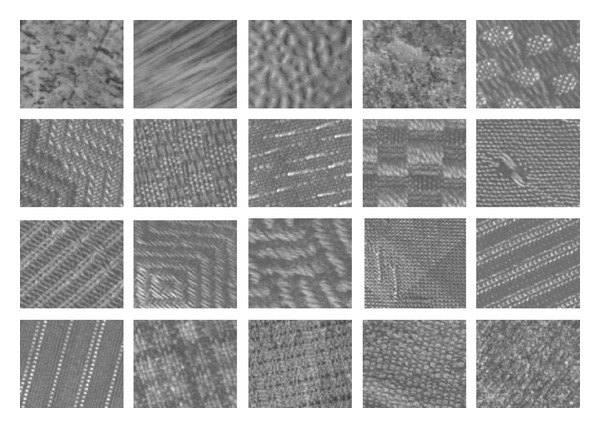
Some images from Outex database.

The Outex datasets (http://www.outex.oulu.fi/index.php?page=classification) include 16 test suites starting from Outex_TC_00010 (TC10) to Outex_TC_00016 (TC16) [31]. These suites were collected under different illumination, rotation, and scaling conditions. Outex_TC_00010 (TC10) and Outex_TC_00012 (TC12) are considered as famous two test suites in Outex datasets. These two suites have the same 24 classes of textures, which were collected under three different illuminates (“horizon,” “inca,” and “t184”) and nine different rotation angles (0°, 5°, 10°, 15°, 30°, 45°, 60°, 75°, and 90°). For each illumination and rotation situation, each class has 20 nonoverlapping texture samples with size of 128 × 128. Examples of Outex images are shown in Figure 7. For TC10, 480 images are used as training data. These are the images of “inca” illumination condition and “0°” angle rotation, whereas the images under the remaining rotation angles and “inca” illumination condition are used as testing data, that is, 3840 images. The training data in case of TC12 is the same as TC10, while all images under “t184” or “horizon” illumination conditions are used as testing data, that is, 4320 images for “t184” and 4320 images for “horizon.” The experimental results of TC10, TC12 (t184), and TC12 (horizon) are shown in Table 1.

From Table 1, the following points can be observed. Firstly, the CLTP_S, CLTP_M, CLTP_M/C, and CLTP_S_M/C performed better than the similar CLBP and CLBC types operators. Secondly, the CLTP_S and CLTP_M showed really great discrimination capability than CLBP_S and CLBP_M, and CLBC_S and CLBC_M, respectively, where the accuracy difference exceeded 10% in some cases. Thirdly, the CLTP_S/M worked well with TC10 and TC12 for (*P* = 1 and *R* = 8), with TC12 for (*P* = 2 and *R* = 16) and only with TC12 under “t184” illumination condition for (*P* = 3 and *R* = 24). Finally, the CLTP_S/M/C achieved the best classification accuracy with TC10 and TC12 for (*P* = 2 and *R* = 16) and TC10 and TC12 under “t184” illumination condition for (*P* = 3 and *R* = 24).

### 4.3. Experimental Results on CUReT Database

**Figure 8:**
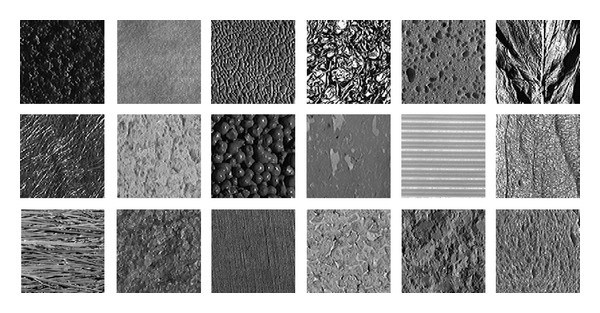
Some images from CUReT dataset.

The CUReT dataset (http://www.robots.ox.ac.uk/~vgg/research/texclass/index.html) has 61 texture classes. In each class, there are 205 images subjected to different illumination and viewpoints conditions [32]. The images in each class have different viewing angle shots. Out of 250 images in each class, there are 118 image shots whose viewing angles are lesser than 60°. Examples of CUReT images are shown in Figure 8. From these types of images, only 92 images are selected after converting to gray scale and cropping to 200 × 200. In each class, *N* images from 92 are used as training data, while the remaining (92 − *N*) are used as testing data. The final classification accuracy is the average percentage over a hundred random splits. The CUReT average classification for *N* = (6,12,23,46) is shown in Table 2. It is easier to note that CLTP has better performance than CLBP and CLBC with the CUReT database. Except CLTP_S/M/C, all CLTP operators are achieving higher classification rates than other CLBP and CLBC for all cases of *N* images and at every radius. The CLTP_S/M/C achieved the best classification rates for all *N* training images at radius 3, for 6, 23, and 64 training images at radius 2 and for 6 training images only at radius 1, while at radius 1, the CLBP_S/M/C achieved the best classification rates for 12, 23, and 64 training images and for 12 training images at radius 2.

### 4.4. Experimental Results on UIUC Database

**Figure 9:**
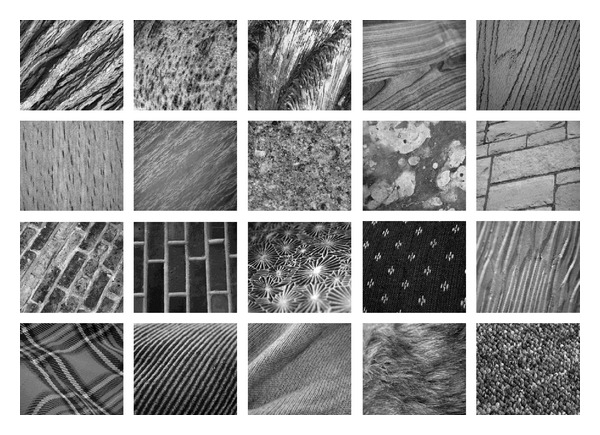
Some images from UIUC database.

The UIUC database has 25 classes containing the images captured under significant viewpoint variations. In each class, there are 40 images with resolution of 640 × 480. Examples of UIUC images are shown in Figure 9. In each class, *N* images from 40 are used as training data, while the remaining (40 − *N*) are used as testing data. The final classification accuracy is the average percentage over a hundred random splits. The UIUC average classification for *N* = (5,10,15,20) is shown in Table 3. Except in case of CLTP_S/M/C, all CLTP operators achieved a higher performance than CLBP and CLBC operators for all *N* number of training images at radiuses 1, 2, and 3. The CLTP_S/M/C outperformed the CLBP_S/M/C and CLBC_S/M/C for all *N* number of training images when *P* = 3, *R* = 24, and *P* = 2, *R* = 16. On the other hand, the CLBC_S/M/C is the best one for small number of *N*, that is, 5 and 10; CLBP_S/M/C is the best one for *N* = 20 and the CLTP_S/M/C is the best one for *N* = 15.

### 4.5. Experimental Results on XU-HR Database

**Figure 10:**
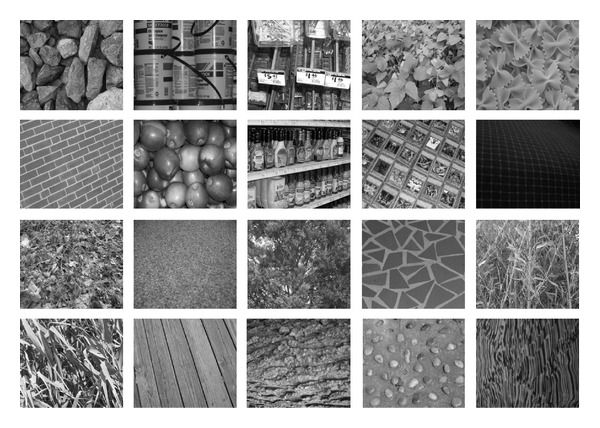
Some images from XU-HR database.

The XU_HR database has 25 classes with 40 high resolution images (1280 × 960) in each class. Examples of XU-HR images are shown in Figure 10. In each class, *N* images from 40 are used as training data, while the remaining (40 − *N*) are used as testing data. The final classification accuracy is the average percentage over a hundred random splits. The XU_HR average classification for *N* = (5,10,15,20) is shown in Table 4. In XU_HR database, the CLTP achieved higher classification rates than CLBP for all *N* training images with all types of radiuses. The CLTP_S/M/C achieved an impressive classification rate reaching 99% when *N* = 20 at (*P* = 3, *R* = 24). We compared this database only with CLBP since HU_HR results using CLBC are not available in [30].

## 5. Conclusion

To overcome some drawbacks of LBP, an existing LTP operator is extended to build a new texture operator, defined as completed Local Ternary Pattern. The proposed associated completed Local Ternary Pattern (CLTP) scheme is evaluated using four challenging texture databases for rotation invariant texture classification. The experimental results in this paper demonstrate the superiority of the proposed CLTP against the new existing texture operators, that is, CLBP and CLBC. This is because the proposed CLTP is insensitive to noise and has a high discriminating property that leads to achieve impressive classification accuracy rates.

## Figures and Tables

**Table 1:** Classification rates (%) on TC10 and TC12 database.

	*R* = 3, *P* = 24				*R* = 2, *P* = 16				*R* = 1, *P* = 8			
	TC10	TC12		Average	TC10	TC12		Average	TC10	TC12		Average
		t184	Horizon			t184	Horizon			t184	Horizon	
CLBP_S	95.07	85.04	80.78	86.96	89.40	82.26	75.20	82.29	84.41	65.46	63.68	71.18
CLBC_S [30]	91.35	83.82	82.75	85.97	88.67	82.57	77.41	82.88	82.94	65.02	63.17	70.38
CLTP_S	98.20	93.59	89.42	93.74	96.95	90.16	86.94	91.35	94.14	75.88	73.96	81.33

CLBP_M	95.52	81.18	78.65	85.12	93.67	73.79	72.40	79.95	81.74	59.30	62.77	67.94
CLBC_M [30]	91.85	75.59	74.58	80.67	92.45	70.35	72.64	78.48	78.96	53.63	58.01	63.53
CLTP_M	98.00	85.39	84.65	89.35	97.32	83.40	84.40	88.37	94.04	75.86	74.05	81.32

CLBP_M/C	98.02	90.74	90.69	93.15	97.44	86.94	90.97	91.78	90.36	72.38	76.66	79.80
CLTP_M/C	98.52	91.23	89.98	93.24	97.94	90.14	92.38	93.49	95.94	84.70	86.02	88.89

CLBP_S_M/C	98.33	94.05	92.40	94.93	98.02	90.99	91.08	93.36	94.53	81.87	82.52	86.31
CLTP_S_M/C	98.98	95.00	92.94	95.64	98.44	92.41	92.80	94.55	96.43	84.00	86.85	89.09

CLBP_S/M	**99.32 **	93.58	93.35	95.42	97.89	90.55	91.11	93.18	94.66	82.75	83.14	86.85
CLBC_S/M [30]	98.70	91.41	90.25	93.45	98.10	89.95	90.42	92.82	95.23	82.13	83.59	86.98
CLTP_S/M	99.04	94.14	92.59	95.26	97.84	92.06	92.69	94.20	96.41	82.85	84.81	88.02

CLBP_S/M/C	98.93	95.32	94.53	96.26	98.72	93.54	93.91	95.39	96.56	** 90.30 **	92.29	93.05
CLBC_S/M/C [30]	98.78	94.00	93.24	95.34	98.54	93.26	94.07	95.29	**97.16 **	89.79	**92.92 **	93.29
CLBC_CLBP [30]	98.96	95.37	**94.72 **	96.35	98.83	93.59	94.26	95.56	96.88	90.25	** 92.92 **	** 93.35 **
DLBPP [30]	98.10	91.60	87.40	92.37	97.70	92.10	88.70	92.83	—	—	—	—
CLTP_S/M/C	99.17	**95.67 **	94.28	**96.37 **	**98.93 **	**94.03 **	**94.79 **	**95.92 **	96.98	87.06	90.30	91.45

The bold values indicate higher classification rate.

**Table 2:** Classification rates (%) on CUReT database.

	*R* = 3, *P* = 24				*R* = 2, *P* = 16				*R* = 1, *P* = 8			
	6	12	23	46	6	12	23	46	6	12	23	46
CLBP_S [28]	66.94	75.26	81.80	87.31	63.49	72.68	79.49	85.35	59.00	67.81	74.62	80.70
CLBC_S [30]	60.82	70.57	74.21	80.14	60.42	68.95	74.42	79.78	56.88	66.21	72.89	78.82
CLTP_S	72.57	81.55	87.72	91.75	68.39	79.09	86.61	91.55	64.38	72.66	81.73	88.24

CLBP_M [28]	62.57	71.93	79.89	86.49	58.57	68.28	76.11	83.03	51.77	60.33	67.73	75.16
CLBC_M [30]	51.23	60.53	68.36	77.41	50.63	58.70	66.05	73.89	50.12	58.62	57.82	66.61
CLTP_M	67.14	76.93	85.16	90.52	63.33	74.47	82.14	88.83	61.37	71.17	80.53	86.67

CLBP_M/C [28]	68.71	78.54	86.04	91.65	64.81	75.56	82.98	89.75	56.53	67.15	75.58	82.97
CLTP_M/C	70.10	80.12	89.02	93.58	66.77	77.12	85.51	91.67	62.07	72.94	82.26	88.98

CLBP_S_M/C [28]	73.29	82.28	89.28	94.07	70.27	80.47	87.57	92.78	66.63	76.54	85.02	90.55
CLTP_S_M/C	74.36	85.14	91.03	94.69	71.55	82.16	87.82	94.04	67.54	78.89	85.46	91.27

CLBP_S/M [28]	74.95	84.30	90.83	94.53	74.63	83.44	89.67	93.85	71.86	82.27	88.57	93.46
CLBC_S/M [30]	70.52	81.57	89.12	93.60	72.16	82.71	89.60	93.78	69.89	79.88	86.62	93.10
CLTP_S/M	76.49	85.11	92.02	95.63	74.14	84.42	90.78	95.06	71.30	82.37	89.20	93.50

CLBP_S/M/C [28]	76.80	86.54	92.00	95.72	76.07	**85.73**	92.15	95.67	74.35	**85.06**	**91.52**	**95.70**
CLBC_S/M/C [30]	73.18	84.07	90.55	95.26	75.17	85.91	91.30	95.39	72.85	82.92	90.12	94.78
CLTP_S/M/C	**77.97**	**87.50**	**92.72**	**96.11**	** 77.72 **	85.54	**92.44**	**95.95**	**75.18**	84.06	90.45	94.78

The bold values indicate higher classification rate.

**Table 3:** Classification rates (%) on UIUC database.

	*R* = 3, *P* = 24				*R* = 2, *P* = 16				*R* = 1, *P* = 8			
	5	10	15	20	5	10	15	20	5	10	15	20
CLBP_S	44.87	54.68	60.63	64.20	41.80	51.34	56.80	60.60	40.05	47.53	51.63	55.29
CLBC_S [30]	47.19	57.46	63.48	66.90	43.37	53.07	59.17	62.39	39.85	46.69	51.11	55.61
CLTP_S	68.80	77.60	83.04	86.00	64.91	75.07	80.48	83.20	54.29	61.87	69.92	71.60

CLBP_M	56.15	65.92	71.05	74.37	56.07	65.65	69.51	72.05	42.39	49.98	54.45	57.52
CLBC_M [30]	51.68	60.62	66.63	69.33	50.67	59.01	64.42	67.10	39.04	45.51	49.42	52.12
CLTP_M	69.94	79.33	82.56	85.20	70.29	79.33	83.36	85.40	57.49	64.67	69.60	73.60

CLBP_M/C	68.08	76.75	80.81	83.27	68.45	76.83	80.14	82.72	56.92	65.09	69.81	72.66
CLTP_M/C	76.80	83.47	87.20	88.60	77.37	83.60	87.04	89.40	70.06	76.93	80.48	81.80

CLBP_S_M/C	69.43	78.61	82.81	85.33	68.68	77.57	81.36	83.55	62.52	71.27	75.48	78.65
CLTP_S_M/C	77.26	84.67	88.48	90.60	77.37	84.27	87.84	89.80	68.80	77.33	80.48	83.60

CLBP_S/M	72.05	82.63	86.88	89.56	71.80	80.85	85.31	87.60	64.70	74.65	79.55	82.58
CLBC_S/M [30]	75.16	83.92	87.68	89.72	73.16	82.04	86.31	88.51	65.28	74.88	78.86	82.40
CLTP_S/M	79.31	87.73	90.56	93.20	77.14	85.60	89.44	91.80	65.03	74.40	79.68	83.00

CLBP_S/M/C	78.05	85.87	89.17	91.07	78.75	86.33	89.25	91.03	74.53	82.26	85.85	**87.86**
CLBC_S/M/C [30]	79.75	86.45	90.10	91.39	79.48	86.63	89.66	91.04	**74.57**	**82.35**	85.66	87.83
CLTP_S/M/C	**82.97**	**88.93**	**91.52**	**94.40**	**82.63**	**87.87**	**90.40**	**92.60**	74.51	81.73	**85.92**	86.80

The bold values indicate higher classification rate.

**Table 4:** Classification rates (%) on XU_HR database.

	*R* = 3, *P* = 24				*R* = 2, *P* = 16				*R* = 1, *P* = 8			
	5	10	15	20	5	10	15	20	5	10	15	20
CLBP_S	75.52	84.27	88.62	91.14	74.11	82.95	86.44	88.77	73.53	81.97	85.91	88.47
CLTP_S	86.06	93.33	95.20	96.60	84.46	90.67	93.60	94.20	76.80	84.13	89.28	91.20

CLBP_M	77.69	85.27	89.07	91.46	77.75	84.99	88.54	90.58	68.13	76.29	80.58	83.64
CLTP_M	86.51	92.13	94.72	96.20	84.23	90.93	93.44	94.80	76.91	84.13	88.32	91.20

CLBP_M/C	85.89	91.29	93.69	95.20	85.80	91.32	93.64	95.07	81.21	87.57	90.40	92.11
CLTP_M/C	91.31	96.27	97.60	97.60	90.29	95.47	96.96	98.00	88.00	92.40	94.72	96.00

CLBP_S_M/C	87.26	92.54	94.67	96.06	87.23	92.66	94.72	95.98	86.37	91.60	93.57	94.65
CLTP_S_M/C	91.89	96.67	97.12	98.20	90.63	95.07	96.80	98.60	87.66	93.20	95.52	96.20

CLBP_S/M	87.09	92.66	94.82	96.10	86.98	92.45	94.67	96.09	84.27	90.24	92.30	93.76
CLTP_S/M	91.77	95.60	97.92	98.60	90.51	95.33	96.96	98.00	84.23	90.40	93.28	94.80

CLBP_S/M/C	91.11	95.33	96.66	97.47	91.64	95.21	96.80	97.50	90.81	94.77	95.80	96.83
CLTP_S/M/C	**95.54**	**98.00**	**98.56**	**99.00**	**94.17**	**97.33**	**98.56**	**98.80**	**91.20**	**95.47**	**96.48**	**97.80**

The bold values indicate higher classification rate.
